# Epstein–Barr virus in thyroid disease: an integrated immunovirological perspective

**DOI:** 10.3389/fimmu.2025.1687214

**Published:** 2025-10-28

**Authors:** Zhengda Wang, Xiwen Chang, Xianbin Cheng

**Affiliations:** ^1^ The Orthopaedic Medical Center, The Second Hospital of Jilin University, Changchun, Jilin, China; ^2^ Joint International Research Laboratory of Ageing Active Strategy and Bionic Health in Northeast Asia of Ministry of Education, Changchun, Jilin, China; ^3^ Department of Breast Surgery, The Second Hospital of Jilin University, Changchun, Jilin, China; ^4^ Department of Thyroid Surgery, The Second Hospital of Jilin University, Jilin, Changchun, China

**Keywords:** Epstein–Barr virus, thyroid disease, immunology, dysregulation, pathogenic mechanism

## Abstract

Epstein–Barr virus (EBV) is a highly prevalent human herpesvirus capable of establishing lifelong latency in the host. It has garnered increasing attention for its potential pathogenic role in thyroid diseases. This review integrates current evidence linking EBV to a spectrum of thyroid diseases, including Graves’ disease, Hashimoto’s thyroiditis, thyroid cancer, and other rare subtypes. A key contribution of this work is the synthesized framework that connects viral infection, immune dysregulation, and neoplastic transformation. Unlike previous studies focused on isolated conditions, this review highlights the bridging role of EBV between autoimmunity and thyroid tumorigenesis. In addition, potential EBV-targeted therapeutic strategies are discussed, offering new perspectives for early diagnosis, risk stratification, and personalized management. Overall, this review advances the mechanistic understanding of EBV-associated thyroid diseases and provides a theoretical foundation for future research and clinical interventions.

## 1 Introduction

Epstein–Barr virus (EBV), classified as human herpesvirus type 4 (HHV-4), is a ubiquitous double-stranded DNA virus with a global adult seroprevalence exceeding 95% (1). Characterized by its pronounced tropism for B lymphocytes, EBV establishes lifelong latency within the host and undergoes periodic reactivation—features that are central to its pathogenic profile (2). Although EBV has traditionally been considered a human-specific pathogen, recent studies using experimental animal models have demonstrated that it can establish cross-species infection under controlled laboratory conditions, such as in rabbits and tree shrews, producing disease manifestations similar to those observed in humans (3, 4). Moreover, investigations in non-human primate and murine models have also confirmed EBV’s ability to establish cross-species infection in experimental settings. These findings underscore the importance of animal models in advancing the understanding of EBV pathogenesis (5, 6). The pathogenic spectrum of EBV is remarkably broad. Beyond its well-established oncogenic roles in malignancies such as nasopharyngeal carcinoma, gastric carcinoma, and Burkitt lymphoma, EBV has also been implicated in the pathogenesis of various autoimmune diseases (7–10). Mechanistically, EBV exerts its effects by expressing a repertoire of viral factors—including latent membrane protein 1 (LMP1), Epstein–Barr nuclear antigens (EBNAs), and small non-coding RNAs (EBERs)—which interfere with host immune regulation. These viral components can modulate antigen presentation, activate proinflammatory signaling cascades, impair immune tolerance, and promote apoptosis, collectively contributing to immune dysregulation and chronic inflammation (1, 11–13). Given EBV’s capacity to manipulate immune responses and disrupt immunological homeostasis, its role in non-neoplastic immunopathology has attracted increasing attention. Elucidating the mechanisms by which EBV persists in tissue-specific microenvironments, reactivates under immunological stress, and interacts dynamically with host immune networks has become a critical focus at the intersection of virology, immunology, and clinical research (7, 14, 15).

In recent years, advances in viral detection technologies and a growing understanding of EBV latency have brought increasing attention to its potential involvement in endocrine disorders, particularly thyroid diseases (16, 17). Accumulating evidence from serological, molecular, and histopathological studies has revealed the presence of EBV-associated signals in patients’ blood, tissues, and cellular environments. These findings suggest that EBV may contribute to the initiation and progression of thyroid pathology through mechanisms such as molecular mimicry, immune evasion, and remodeling of the local inflammatory microenvironment (18–21). However, an integrated overview of EBV’s role across thyroid diseases remains lacking. In particular, there is a gap in understanding the longitudinal mechanisms linking early-stage inflammation to malignant transformation. This review consolidates recent advances in the investigation of EBV-thyroid interactions, with emphasis on key dimensions such as virological detection, latent infection patterns, immune regulatory mechanisms, and activation of downstream signaling pathways. A mechanistic framework is proposed to outline the EBV-mediated continuum of thyroid disease, with implications for early diagnosis, biomarker development, and personalized interventions. To ensure methodological transparency, we systematically searched PubMed up to August 2025 using the terms (“Epstein–Barr virus” OR “EBV”) AND (“thyroid” OR “Graves’ disease” OR “Hashimoto’s thyroiditis” OR “thyroid cancer” OR “autoimmune thyroid disease”). We included peer-reviewed original articles, case reports, and reviews that provided clinical, molecular, or immunological evidence on EBV–thyroid interactions. Non-English publications, conference abstracts, and studies lacking primary data were excluded. This approach ensured a comprehensive and unbiased integration of current evidence.

## 2 The role of EBV in thyroid diseases

### 2.1 EBV and Graves’ disease

Graves’ disease (GD) is the most prevalent cause of hyperthyroidism worldwide, with an estimated prevalence of 3% in women and 0.5% in men (22, 23). As an organ-specific autoimmune disorder, GD is primarily characterized by the aberrant production of thyroid-stimulating hormone receptor antibodies (TRAbs) (24–26). These autoantibodies chronically stimulate the thyroid-stimulating hormone (TSH) receptor expressed on thyroid follicular epithelial cells, leading to excessive synthesis and secretion of thyroid hormones. This hyperthyroid state manifests clinically as a constellation of high metabolic symptoms, including palpitations, weight loss, heat intolerance, and ophthalmopathy (27–29). The pathogenesis of GD is currently understood as the result of a complex interplay among genetic susceptibility, immune dysregulation, endocrine imbalances, and environmental triggers (30–32). Among environmental factors, viral infections, particularly those capable of establishing latent or persistent infections, such as EBV, have been implicated in both the initiation and maintenance of various autoimmune processes (33, 34). A comprehensive examination of the relationship between EBV and GD, especially regarding the virus’s role in initiating thyroid-specific autoimmunity, promoting B cell–mediated TRAb production, and modulating disease progression through latent infection or reactivation, may provide novel insights into the immunopathogenesis of GD.

#### 2.1.1 Serological evidence of EBV in Graves’ disease

Nagata et al. performed serological analyses in a cohort of 66 GD patients and 32 healthy controls, revealing a markedly elevated seroprevalence of antibodies against EBV early antigen (EA) in GD patients (p<0.0001). Among individuals with TRAb levels ≥10%, EA antibody titers showed a significant positive correlation with TRAb concentrations (rs=0.443, p=0.0037), suggesting that EBV reactivation may enhance TRAb production through stimulation of autoreactive B cells. In addition, EA antibody levels were positively associated with serum immunoglobulin E (IgE) concentrations (rs=0.318, p=0.0371), pointing to a broader role of EBV in immunoglobulin class switching and systemic immune activation (35). Consistent serological associations reported in other independent investigations further reinforce the credibility of EBV as a potential environmental contributor to GD pathogenesis (36, 37).

#### 2.1.2 Tissue localization evidence of EBV in Graves’ disease

Janegova et al. conducted EBV-associated molecular analyses in thyroid tissues from GD patients and reported that 62.5% of samples exhibited positive expression of EBERs, predominantly localized to thyroid follicular epithelial cells. In contrast, no EBV-positive signals were detected in nodular goiter tissues from control subjects, suggesting a potential disease-specific role for EBV infection in GD pathogenesis (38). Importantly, direct histological evidence of EBV persistence was also observed in the thyroid tissue of one GD patient, where EBER1-positive lymphocytic infiltration was confirmed via *in situ* hybridization. Furthermore, Pyzik et al. employed quantitative real-time PCR to detect EBV DNA in peripheral blood mononuclear cells (PBMCs). EBV DNA was detected in 35.9% (14/39) of GD patients, whereas none of the healthy controls (0/20) tested positive (p=0.01) (39). Notably, the EBV positivity rate was significantly higher in female patients than in males, suggesting a sex-related influence on the contribution of EBV to GD pathogenesis. Collectively, these findings indicate that EBV persists within the thyroid microenvironment and may act as a cofactor in the immune dysregulation and clinical exacerbation of GD, particularly in patients with elevated TRAb titers. Although these studies suggest an association between EBV and GD, the reported positivity rates differ widely. This variability likely reflects differences in detection methods, study populations, and disease stage, underscoring the need for standardized approaches. Moreover, not all studies have consistently confirmed the presence of EBV in GD tissues. Vrbikova et al. conducted *in situ* hybridization on thyroid tissues from GD patients but failed to detect significant EBV-related signals compared with nodular goiter controls, suggesting that the association may not be universal or could depend on specific patient subtypes or detection thresholds (40). A detailed summary of these findings is presented in **Table 1**.

**Table 1:** Summary of EBV detection methods, positivity rates, and proposed pathogenic mechanisms in graves’ disease.

Detection method	Region sample size	EBV positivity rate	Proposed pathogenic mechanism	Reference
EBER in situ hybridization	Slovak Republic N=42	62.5% of GD patients were EBER positive	EBV latent infection in thyroid follicular epithelial cells and B cells induces TRAb production and local immune activation; reactivation further amplifies immunity, contributing to Graves’ disease pathogenesis.	(38)
PCR detection of EBV DNA	Poland N=59	30.8% of GD patients were EBV positive		(39)
	Japan N=98	60% of GD patients were EBV positive		(35)
Detection of EBV EA IgG antibodies in peripheral blood	Japan N=3	61.5% of GD patients were EA IgG positive		(42)
Flow cytometric detection of TRAb^+^EBV^+^ B cells	Japan N=24	43.5% of GD patients had TRAb^+^EBV^+^ B cells		(43)
	Japan N=15	TRAb^+^EBV^+^ B cells were detected in all GD patients		(50)
ELISA detection of EBV-specific antibodies	Japan N=98	96.8% of GD patients were EBV IgG positive	EBV infection acts as an environmental trigger, inducing autoimmune responses	(47)
	Japan N=49	91.2% of GD patients were EBV IgG positive	EBV infection contributes to GD pathogenesis by activating B cells and inflammatory pathways	(48)
	Japan N=24	94.7% of GD patients were EBV IgG positive	EBV-induced chronic B cell activation may contribute to the development of autoimmunity	(49)
RT-qPCR detection of LMP1 mRNA expression	Japan N=38	31.1% of GD patients were LMP1 positive	LMP1 expression enhances B cell activity and promotes autoantibody production	(53)

#### 2.1.3 Mechanisms of EBV in Graves’ disease progression

**Figure 1:**
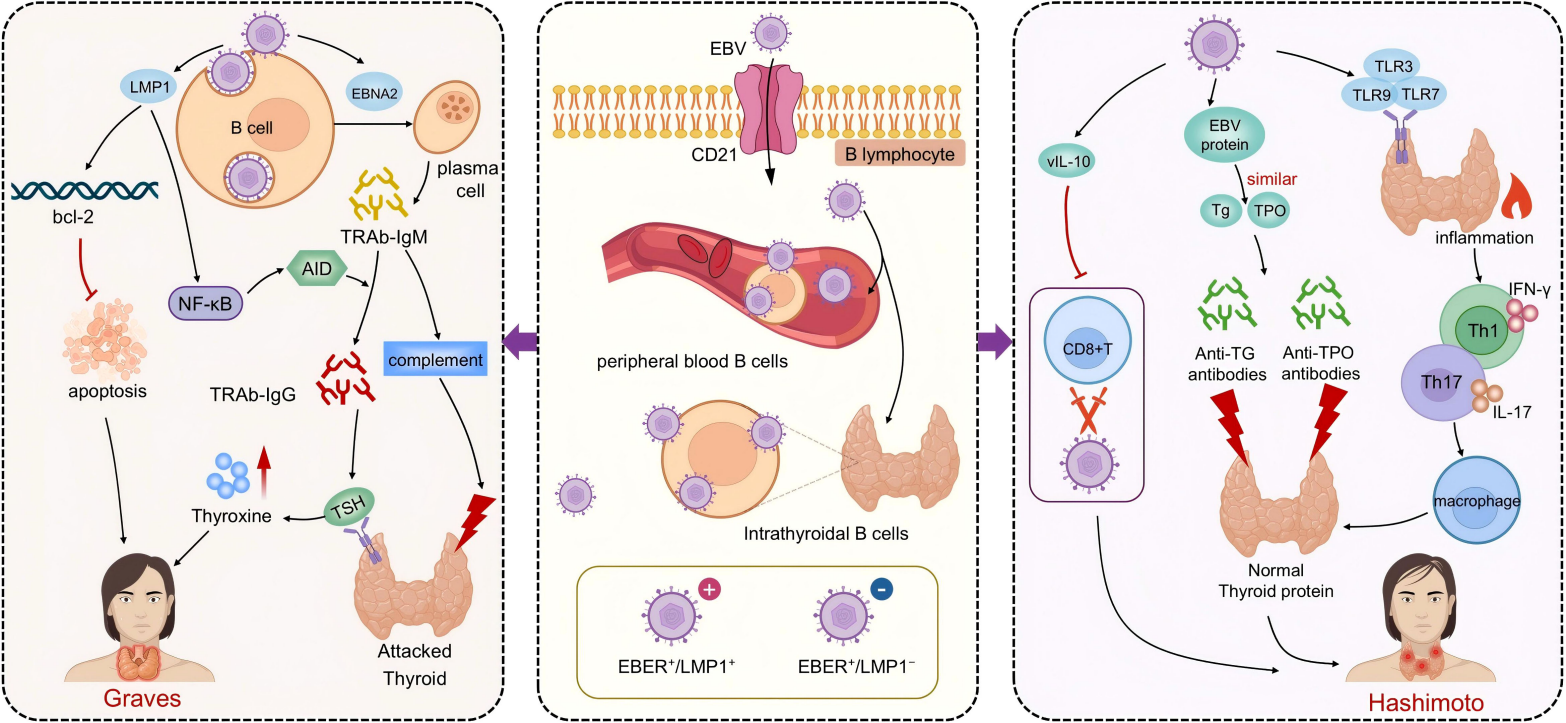
The pathogenic mechanisms of EBV in Graves’ disease and Hashimoto’s thyroiditis. The central portion of the figure highlights EBV infection of B lymphocytes via the CD21 receptor, with latency established both in peripheral blood and thyroid-infiltrating B cells. The left panel depicts EBV-related mechanisms in Graves’ disease (GD). EBV promotes the differentiation of B cells into TRAb-producing plasma cells. LMP1 mimics CD40 signaling, activates the NF-κB pathway, prolongs B cell survival, and facilitates class switching to generate TRAb-IgG and IgM. TRAb-IgG drives TSH receptor overstimulation and thyroid hormone overproduction, while TRAb-IgM induces complement-mediated cytotoxicity, contributing to GD pathogenesis. The right panel illustrates EBV’s role in Hashimoto’s thyroiditis (HT). Viral proteins mimic thyroid autoantigens, triggering immune cross-reactivity and production of anti-Tg and anti-TPO antibodies. EBV also activates TLR pathways, induces Th1/Th17-mediated inflammation, and secretes vIL-10 to impair CD8^+^ T cell–mediated clearance, sustaining chronic inflammation and thyroid dysfunction characteristic of HT.

EBV infection has been proposed as a potential environmental trigger in the development of GD (**Figure 1**). A notable case involved a 6-year-old girl who developed transient hyperthyroidism following primary EBV infection. Laboratory evaluation revealed a significant decrease in serum TSH levels alongside elevated concentrations of free triiodothyronine (FT3) and free thyroxine (FT4), consistent with clinical hyperthyroidism. Concurrently, high titers of anti-thyroid peroxidase and anti-thyroglobulin antibodies were detected. Based on the temporal association with acute EBV infection, biochemical thyroid dysfunction, and serological autoantibody profile, the condition was diagnosed as EBV-induced thyrotoxicosis (41). Additional evidence was reported by Akahori et al., who described three cases in which patients developed overt GD during the acute phase of EBV infection. These individuals exhibited hallmark features of thyrotoxicosis, including suppressed TSH, elevated thyroid hormone levels, and positive TRAb. Notably, these clinical and immunological features were observed, suggesting that EBV infection alone may be sufficient to disrupt immune tolerance and initiate thyroid-specific autoimmunity, thereby contributing to GD onset (42).

Nagata et al. were the first to identify a population of B lymphocytes co-expressing TRAbs and EBV markers in the peripheral blood of patients with GD using flow cytometry combined with confocal laser scanning microscopy. The frequency of these dual-positive cells was significantly higher in GD patients compared to healthy controls, suggesting that EBV may promote TRAb production by infecting autoreactive B cells and thereby contribute to disease initiation (43). In a subsequent study involving a pediatric case of infectious mononucleosis induced by primary EBV infection, the researchers observed that the appearance of TRAbs during the acute phase coincided with the expression of both lytic and latent EBV genes. This temporal association implies that EBV not only activates the immune system but may also directly trigger the generation of autoantibodies. EBNA2, a critical transcriptional activator in B cells, can drive their differentiation into antibody-secreting plasma cells, thereby amplifying humoral responses and potentially accelerating GD pathogenesis (44). In more recent work, the same group focused on TRAb-producing B cells of the IgM isotype and found that these cells often harbored latent EBV infection and expressed co-stimulatory molecules such as cluster of differentiation (CD) 40 and CD80, indicative of antigen-presenting capacity and immunological activation. Although IgM-type TRAbs lack classical TSH receptor agonist activity, they may mediate complement-dependent cytotoxicity, leading to damage of thyroid follicular cells, particularly during the early inflammatory phase or disease relapse (45, 46). Kumata et al. further confirmed that TRAbs in the serum of GD patients are predominantly of the IgM subclass, and TRAb-IgM levels were significantly elevated in individuals with high EBV antibody titers. This finding supports the hypothesis that EBV reactivation drives the differentiation of autoreactive B cells into plasma cells capable of secreting TRAb-IgM. LMP1 can stimulate B cells while upregulating anti-apoptotic factors such as Bcl-2, enabling the survival of autoreactive IgM^+^ B cells that would otherwise be eliminated through tolerance mechanisms. These cells may persist and contribute to autoantibody production through mechanisms involving immune evasion and non-specific activation (47). Furthermore, LMP1 has been shown to activate the NF-κB signaling pathway. This activation subsequently upregulates the expression of activation-induced cytidine deaminase, an essential enzyme required for immunoglobulin class-switch recombination. This process enables the transition of B cells from producing low-affinity IgM to high-affinity IgG autoantibodies, including pathogenic TRAbs, thereby facilitating disease progression in GD (48–50).

#### 2.1.4 Latency mechanisms of EBV in Graves’ disease

EBV exhibits an exceptionally high seroprevalence in adult populations and is typically maintained in a latent state within B lymphocytes. Notably, in patients with GD, EBV also predominantly persists in a latent rather than lytic or actively replicating form. Although elevated levels of EBV-specific antibodies and, in some cases, detectable EBV DNA have been observed in thyroid tissue or peripheral blood of GD patients, the direct detection of viral genomic material within thyroid tissue remains rare. These findings suggest that EBV is more likely to exist in a silent, latent infection state in GD, rather than as an active or acute infection. Its contribution to disease pathogenesis may instead occur through indirect mechanisms such as chronic immune activation or molecular mimicry (51). Supporting this notion, Pyzik et al. reported a significantly higher prevalence of EBV DNA in peripheral blood mononuclear cells (PBMCs) from GD patients compared to healthy controls (52). However, markers indicative of viral reactivation were not consistently elevated, further reinforcing the interpretation that EBV infection in GD is predominantly latent and of low replicative activity.

An EBER^+^/LMP1^-^ phenotype is indicative of a quiescent latent EBV infection, whereas an EBER^+^/LMP1^+^ profile reflects a more active latent state with potential for cellular transformation. Several histopathological studies have reported high expression levels of EBERs in the thyroid tissues of patients with GD. However, LMP1 was largely undetectable in these tissues, suggesting that EBV predominantly exists in a silent or non-replicative form, with minimal direct involvement in tissue damage (38, 53). Despite the rarity of active viral replication in GD-associated tissues, latent or low-level reactivation of EBV may still contribute to disease onset and persistence through indirect immunological mechanisms. For instance, viral proteins may engage in molecular mimicry by structurally resembling thyroid autoantigens, thereby triggering cross-reactive T or B cell responses and breaking immune tolerance. Alternatively, EBV may amplify local immune responses through the “bystander effect”, activating nonspecific B cells within an inflamed microenvironment and expanding the autoimmune repertoire. In addition, EBV-derived components can activate innate immune sensors such as toll-like receptors (TLR), leading to downstream release of pro-inflammatory cytokines and further disruption of immune homeostasis. Therefore, although EBV in GD most commonly exists in a latent state, its reactivation under specific conditions such as hormonal fluctuations, immunosuppression, or concurrent infections may stimulate antibody production and immune activation. This could help explain the heterogeneity and relapsing-remitting nature of GD observed in clinical practice (54, 55).

### 2.2 EBV and Hashimoto’s thyroiditis

Hashimoto’s thyroiditis (HT) is the most prevalent organ-specific autoimmune thyroid disorder and a leading cause of hypothyroidism worldwide (56, 57). Its pathogenesis is thought to arise from the interplay between genetic susceptibility and environmental triggers, culminating in the breakdown of immune tolerance, the sustained production of thyroid-specific autoantibodies, and chronic inflammatory destruction of thyroid follicles (58, 59). In recent years, increasing attention has been directed toward the role of viral infections in the pathogenesis of HT. Particular focus has been placed on viruses that exhibit latency and the capacity for reactivation, given their potential to disrupt immune tolerance and trigger autoimmune responses (60). Among these, EBV has garnered interest due to its well-established association with various autoimmune diseases. Accumulating evidence indicates elevated levels of EBV-related latent transcripts, viral antigens, and specific antibodies in both the peripheral blood and thyroid tissues of HT patients. These findings suggest that EBV may persist or undergo localized reactivation within the thyroid, thereby triggering aberrant immune responses, disrupting self-tolerance, and contributing to the immunopathological cascade of HT. Elucidating the pathogenic potential of EBV in HT may not only provide mechanistic insights into disease etiology but also offer valuable markers for identifying high-risk individuals and inform preventive strategies. Moreover, this line of research may pave the way for the development of targeted antiviral therapeutic approaches in the management of virus-associated thyroid autoimmunity (61–63).

#### 2.2.1 Serological evidence of EBV in Hashimoto’s thyroiditis

Several studies have systematically evaluated the serological profile of EBV infection in patients with HT and consistently reported significantly higher seropositivity rates and antibody titers compared to healthy controls. This elevation was observed across both younger and older subgroups (<40 and ≥40 years), with statistically significant differences (p<0.01), suggesting a possible state of chronic EBV infection or viral reactivation in HT patients (40, 64). A population-based case–control study further confirmed that HT patients exhibited markedly elevated levels of EBV-specific antibodies, particularly viral capsid antigen IgG and early antigen IgG, with serum concentrations significantly higher than those in matched healthy individuals (p=0.002 and p=0.001). These findings imply that both prior EBV exposure and reactivation events may be associated with HT pathogenesis. Moreover, EBNA1 IgG levels showed significant correlations with thyroid functional parameters such as FT3, TSH, and anatomical features like isthmus thickness, indicating a potential involvement of EBV in thyroid functional dysregulation. Interestingly, even within the control group, EBNA1 IgG levels positively correlated with anti-thyroid peroxidase antibodies, suggesting that EBV may play a role in the early immunological activation phase of HT (65). Another population-based study demonstrated an enhanced EBV-specific immune response in HT patients. Notably, the seropositivity rate of EBNA1 antibodies reached 52% in the HT group, significantly higher than the 30% observed in healthy controls. Additionally, increased early antigen-diffuse antibody positivity suggested potential reactivation of latent EBV infection, further supporting the hypothesis that EBV contributes to both the initiation and progression of HT (66).

#### 2.2.2 Tissue localization evidence of EBV in Hashimoto’s thyroiditis

Evidence of EBV presence in HT has also been demonstrated at the tissue and molecular levels. Janegova et al. examined thyroid tissues from HT patients using immunohistochemistry and *in situ* hybridization techniques. Their analysis revealed EBERs expression in 80.7% of HT samples and cytoplasmic expression of LMP1 in 34.5% of cases, primarily localized to follicular epithelial cells and infiltrating lymphocytes. In contrast, no EBV-positive staining was observed in control tissues from patients with nodular goiter (38). The EBER^+^/LMP1^-^ phenotype indicates a quiescent latent infection, whereas EBER^+^/LMP1^+^ expression suggests a more active latent state with potential for cellular transformation. These findings support the notion that EBV persists in HT lesions predominantly in a latent form. Additionally, a population-based study involving 129 paraffin-embedded thyroid tissue samples from HT patients detected the LMP1 gene via PCR in 11.1% of cases, further suggesting the possibility of persistent EBV latency in the thyroid tissue of a subset of HT patients (67). While EBV-related signals are frequently observed in HT, the positivity rates vary considerably across cohorts. Such heterogeneity may arise from methodological differences, population characteristics, and disease heterogeneity, highlighting the importance of harmonized methodologies. A detailed summary illustrating the involvement of EBV in HT is presented in **Table 2**.

**Table 2:** Summary of EBV detection methods, positivity rates, and proposed pathogenic mechanisms in hashimoto’s thyroiditis.

Detection method	Region sample size	EBV positivity rate	Proposed pathogenic mechanism	Reference
LMP1 immunohistochemistry, EBER in situ hybridization	Slovak Republic N=42	34.5% of HT patients tested positive for LMP1, while 80.7% were EBER-positive	EBV latent infection and reactivation can activate inflammatory responses, promote immune cell infiltration, and collectively contribute to the chronic inflammatory process in HT by inducing immune imbalance and autoantibody production.	(38)
RT-qPCR for detection of LMP1 mRNA	Japan N=38	27.3% of HT patients showed LMP1 positivity		(53)
ELISA for detection of EBV-specific antibodies	Sudan N=135	95.2% of HT patients were EBV VCA-IgG positive, 57.1% were EA-IgG positive, and 90.5% were EBNA-IgG positive	High-titer EBV antibodies suggest persistent viral infection and immune activation, which may collectively contribute to the pathogenesis of HT through B-cell involvement and inflammatory responses.	(67)
IFA for detection of EBV-specific antibodies	Czech Republic N=57	81.8% of HT patients exhibited IgG titers ≥1:320		(40)
EBER in situ hybridization	Egypt N=120	42.3% of HT patients were EBER-positive	EBV establishes latent infection in thyroid follicular epithelial cells, activating local inflammatory and immune responses that may trigger the development of autoimmune thyroiditis.	(65)
	Poland N=25	48% of HT patients were EBER-positive		(70)
Detection of EBV-specific antibodies by IgM immunoblot	India N=109	52% of HT patients were EBNA-1 IgG positive	EBV reactivation induces antigen expression and activates immune responses, with elevated EBNA-1 antibody levels indicating either latent or reactivated infection status.	(66)

#### 2.2.3 Mechanisms of EBV in Hashimoto’s thyroiditis progression

Current evidence suggests that EBV may contribute to the development and progression of HT through multiple mechanisms beyond latency, including molecular mimicry, immune evasion, and activation of innate immune responses (**Figure 1**). Among these, molecular mimicry is considered one of the most critical pathways. EBV antigens share structural homology with thyroid-specific self-antigens, such as thyroid peroxidase and thyroglobulin, leading the host immune system to misidentify thyroid tissue as foreign. This cross-reactivity promotes the generation of autoantibodies, including anti-thyroid peroxidase and anti-Tg antibodies. Elevated levels of EBV-related antibodies, such as viral capsid antigen and EBNA, observed in HT patients, further support this mechanism (65, 68, 69). Immune cell dysfunction has also been linked to EBV-related pathogenesis in HT. Studies have reported a reduced number of EBV-specific CD8^+^ T cells and an elevated CD4/CD8 ratio, suggesting impaired cytotoxic responses against EBV-infected B cells. This weakened viral clearance may allow persistent antigenic stimulation, thereby exacerbating autoimmune responses (21). Additionally, EBV can disrupt innate immune balance by activating Toll-like receptor signaling pathways. One study found that nearly half of newly diagnosed HT patients tested positive for EBV DNA and exhibited significantly increased expression of TLR3, TLR7, TLR8, and TLR9 in both T and B lymphocytes. Serum levels of soluble TLRs were also elevated. These findings suggest that EBV may bind to TLR9 via its viral DNA, initiating CpG DNA-mediated inflammatory signaling. Simultaneous activation of TLR7 and TLR8 in both B and T cells could further amplify the release of pro-inflammatory mediators, intensifying immune dysregulation in HT (70). Collectively, these disease mechanisms illustrate that EBV not only persists latently but also actively shapes the immune landscape through molecular mimicry, impaired clearance, and TLR-driven inflammation, thereby driving thyroid-specific autoimmunity.

#### 2.2.4 Latency mechanisms of EBV in Hashimoto’s thyroiditis

EBV is capable of establishing long-term latency in B lymphocytes, with its viral genome persisting within host cells and undergoing periodic reactivation. This latent state may sustain a chronic inflammatory environment that contributes to HT progression. Latency-associated proteins such as LMP1 and EBERs have been detected in the thyroid tissues of HT patients, where they activate the NF-κB signaling pathway, trigger proinflammatory cascades, and impair immune tolerance, thereby promoting disease exacerbation (69). Moreover, EBV encodes Bcl-2 homologs with anti-apoptotic functions, prolonging the survival of infected B cells, and produces a viral interleukin-10 homolog that suppresses antiviral immune responses and impairs immune-mediated clearance of infected cells. These latency-related strategies facilitate viral persistence and contribute to local immune dysregulation in the thyroid microenvironment. Supporting this, Li et al. proposed that EBV enters B cells by binding its envelope glycoprotein gp350 to the CD21 receptor. During the latent infection phase, the expression of viral proteins such as EBNA and LMP is induced. These latency-associated proteins can impair B cell function, prolong survival, and create conditions favorable for immune escape (71).

### 2.3 EBV and thyroid cancer

In recent years, the incidence of thyroid cancer has shown a continuous upward trend, making it one of the most common malignancies of the endocrine system. Among its histological subtypes, papillary thyroid carcinoma (PTC) is the most prevalent, accounting for approximately 80% of all cases, followed by follicular thyroid carcinoma (FTC), anaplastic thyroid carcinoma (ATC), and medullary thyroid carcinoma (MTC) (72–75). Although PTC is generally associated with a favorable prognosis, a subset of patients may experience local recurrence, aggressive growth, or distant metastasis, indicating that its pathogenesis is influenced by both intrinsic molecular mechanisms and external environmental factors (76–78). EBV has long been implicated in the development of various malignancies, including nasopharyngeal carcinoma, gastric cancer, and multiple lymphoproliferative disorders (13, 79). More recently, the potential oncogenic role of EBV in thyroid cancer has garnered increasing scientific interest (80, 81). A systematic investigation of the relationship between EBV and thyroid cancer may provide novel insights into the virus’s contribution to tumor initiation and progression. Such research could also offer a theoretical basis for improving early detection, identifying relevant biomarkers, and developing targeted therapeutic strategies for EBV-associated thyroid malignancies.

#### 2.3.1 Serological evidence of EBV in thyroid cancer

Epidemiological studies have demonstrated a considerable prevalence of EBV in thyroid cancer patients across diverse populations. Among the histological subtypes of thyroid cancer, PTC is the most prevalent, accounting for approximately 80–85% of all cases. The reported EBV detection rates in PTC vary considerably across populations, ranging from 47–71% in the study by Wu et al., with significantly higher prevalence in Asian cohorts compared to Western populations, to 65.9% in an Iranian cohort and only 15.8% in a Brazilian cohort (82). FTC, which represents about 10–15% of thyroid cancers, has shown no evidence of EBV positivity in large cohorts such as the Guangdong study (83). MTC, accounting for 3–5% of cases, has similarly tested negative for EBV in large-scale investigations. This inconsistency is likely due to differences in detection methods, the possibility of lymphoid infiltration leading to false positives, and population-specific factors such as genetic background or viral strain variation. These issues highlight the need for standardized and carefully controlled approaches when assessing EBV prevalence in thyroid carcinoma. Although ATC constitutes less than 2% of thyroid cancers, elevated expression of EBV-encoded proteins such as EBNA2 and LMP1 has been observed, suggesting a potential role for EBV in the dedifferentiation from PTC to ATC and in promoting tumor aggressiveness (84). This dedifferentiation refers to the pathological progression of PTC into ATC, during which EBV-encoded proteins such as LMP1 and EBNA2 facilitate epithelial–mesenchymal transition, anti-apoptotic signaling, and immune evasion, thereby driving tumor aggressiveness. This regional difference suggests that the oncogenic potential of EBV may be influenced by host genetic background, environmental exposures, lifestyle, or viral strain diversity. Moreover, in a cohort of 41 PTC patients, 27 were EBNA1-positive, with younger patients (<30 years old) displaying a higher positivity rate, indicating a potential age-related susceptibility to EBV-driven oncogenesis (85). Collectively, these serological and PCR-based findings underscore the potential contribution of EBV to thyroid tumor development and progression in specific demographic subgroups.

#### 2.3.2 Tissue localization evidence of EBV in thyroid cancer

The localization of EBV within thyroid tumor tissues has been extensively investigated using molecular and immunohistochemical approaches. Techniques such as mRNA *in situ* hybridization, immunofluorescence staining, and PCR have consistently identified EBV in thyroid cancer specimens across all histological subtypes, independent of the degree of differentiation (86, 87). Notably, an elevated expression of EBV-encoded oncogenic proteins, including EBNA2 and LMP1, has been observed in ATC samples, implicating EBV in the dedifferentiation process from PTC to ATC. Increased EBV expression has also been correlated with higher tumor aggressiveness and poorer clinical outcomes, particularly in undifferentiated thyroid cancers (88). Supporting these findings, Takahashi et al. detected both Z Epstein–Barr replication activator (ZEBRA) protein and EBER expression in thyroid malignant lymphomas, further strengthening the evidence for EBV involvement in thyroid-derived malignancies (89). Additionally, higher EBV DNA loads have been observed in thyroid cancer tissues compared to adjacent normal tissues, providing compelling molecular evidence for its role in malignant transformation (85). A detailed summary illustrating the involvement of EBV in thyroid cancer is presented in **Table 3**.

**Table 3:** Summary of EBV detection methods, positivity rates, and proposed pathogenic mechanisms in thyroid cancer.

Detection method	Region sample size	EBV positivity Rate	Proposed pathogenic mechanism	Reference
In situ hybridization for EBER	Japan N=22	51.6% of TC patients were EBER-positive, including 47.4% in classical PTC, 66.6% in follicular variant PTC, and cases with undifferentiated carcinoma.	EBV infection is widely distributed in tumor and surrounding tissues, and through latent infection of epithelial cells and tumor-associated lymphocytes, it activates local inflammatory pathways and immune responses, modulates the tumor immune microenvironment, and collectively promotes the development and progression of thyroid cancer.	(88)
	Finland N=315665	9.8% of TC patients were EBER-positive.		(92)
	Portugal N=3	13.9% of TC patients were EBER-positive.		(94)
	Iran N=75	55.9% of TC patients were EBER-positive.		(95)
	Japan N=41	62.5% of TC patients were EBER-positive.		(99)
	Turkey N=81	58.1% of TC patients were EBER-positive, with 16.1% positivity in tumor-infiltrating lymphocytes and 53.2% in adjacent normal follicular epithelial cells.		(100)
PCR detection of the EBNA-1 gene	Iran N=41	65.8% of TC patients were EBNA-1-positive.	EBV promotes carcinogenesis by affecting oncogenes such as BRAF through EBNA-1.	(85)
PCR detection of EBV DNA	Iran N=88	71.9% of TC patients were EBV DNA-positive.	EBV contributes to tumor progression by infecting follicular cells or tumor-infiltrating lymphocytes, inducing chronic inflammation and immune activation.	(90)

#### 2.3.3 Mechanisms of EBV in thyroid cancer progression

**Figure 2:**
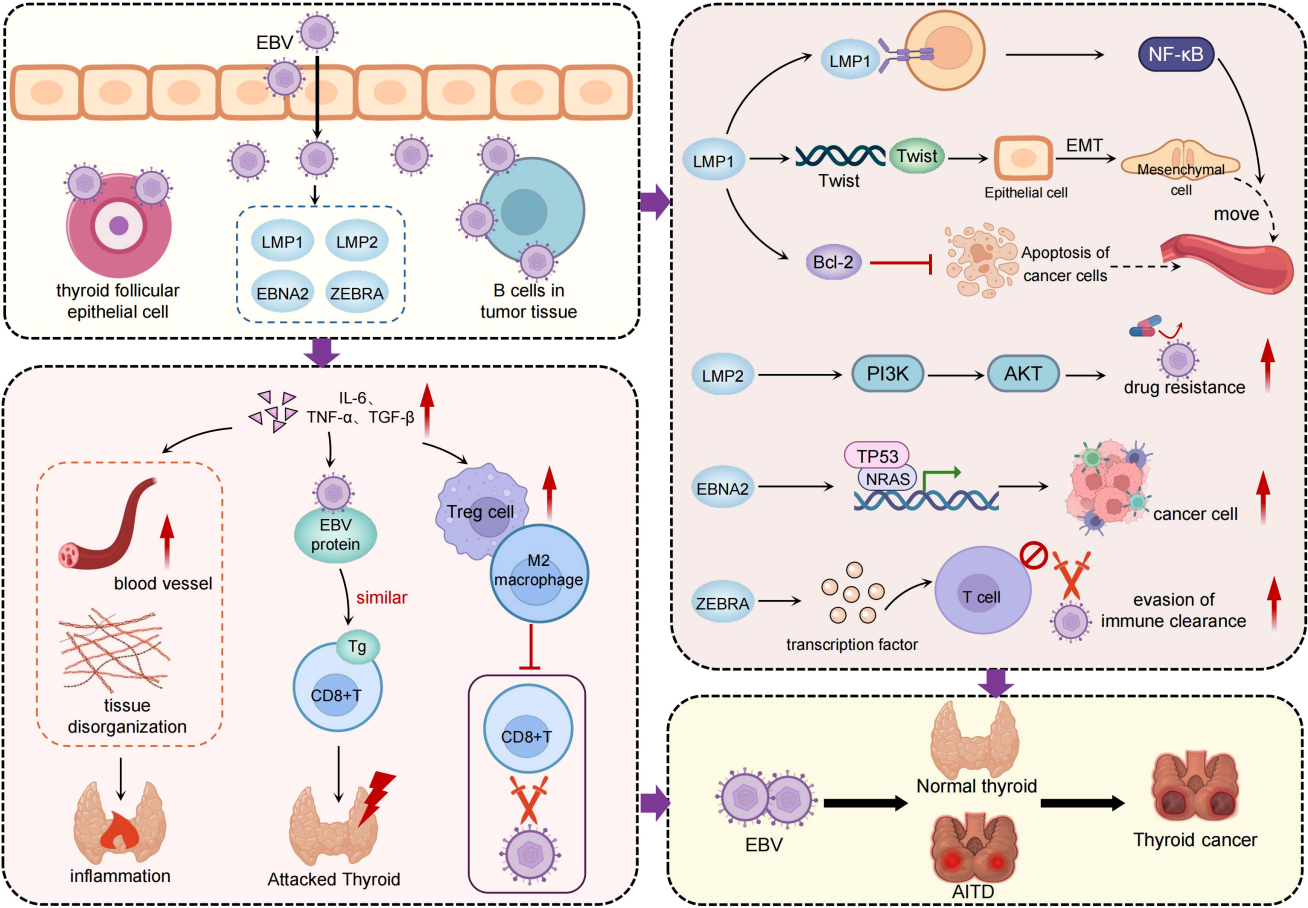
The pathogenic mechanisms of EBV in thyroid cancer. The central portion of the figure highlights EBV infection of thyroid follicular epithelial cells and tumor-infiltrating B cells, with high expression of viral markers such as EBER, EBNA1, LMP1, and LMP2 detected in thyroid cancer tissues. The left panel illustrates EBV-mediated remodeling of the tumor microenvironment. By inducing inflammatory cytokines such as IL-6, TNF-α, and TGF-β, EBV promotes chronic inflammation and facilitates the infiltration of regulatory T cells and M2-polarized macrophages. This immunosuppressive environment weakens host immune surveillance and contributes to tissue disorganization. The right panel depicts how EBV activates key oncogenic pathways through its latent and lytic proteins. LMP1 stimulates NF-κB signaling and upregulates Twist, driving epithelial–mesenchymal transition and enhancing tumor invasiveness. LMP2 activates the PI3K/AKT pathway, promoting cell survival and drug resistance. EBNA2 interferes with tumor suppressor genes such as TP53 and NRAS, contributing to tumor dedifferentiation. ZEBRA impairs T cell activation, facilitating immune evasion. In addition, EBV may promote the transition from autoimmune thyroid diseases to thyroid cancer, underscoring its bridging role in inflammation-driven tumorigenesis.

EBV contributes to the initiation and progression of thyroid cancer through multiple molecular mechanisms involving its LMP1, LMP2 and EBNAs (**Figure 2**). LMP1 mimics CD40 receptor activation, thereby inducing the expression of cyclins and promoting tumor cell proliferation. In parallel, LMP2 enhances cellular survival and drug resistance by activating the PI3K/Akt signaling pathway. These findings suggest that EBV not only induces malignant transformation through direct infection but also remodels the tumor microenvironment to promote immune modulation, invasion, and therapeutic resistance in thyroid carcinoma cells (90). During latent infection, the roles of LMP1 and EBNA2 are particularly critical. LMP1 promotes epithelial to mesenchymal transition, which enhances tumor cell invasiveness and metastatic potential. EBNA2 has been implicated in regulating tumor aggressiveness and facilitating cancer dissemination. In the context of viral latency and immune evasion, EBV activates the expression of viral genes such as LMPs and EBNAs, which suppress host antitumor immune responses. This enables the virus to evade immune surveillance and facilitates tumor development and progression. Moreover, EBV infection has been shown to activate anti-apoptotic pathways. It upregulates the expression of key survival-related proteins such as survivin, Bcl-2, CD44, and NF-κB. This inhibits programmed cell death and enhances cancer cell viability and proliferation. These molecular alterations collectively contribute to the aggressive behavior and poor prognosis observed in EBV-associated thyroid malignancies (91).

Chronic inflammation and immune microenvironmental alterations represent key mechanisms through which EBV contributes to thyroid cancer progression. EBV infection may induce persistent inflammatory responses that reshape the immune landscape of thyroid tumors. Studies have demonstrated that EBV, once present within thyroid cancer tissues, can interact with immune cells to modulate tumor immune evasion and alter the tumor microenvironment (86). Tumor dedifferentiation is another important mechanism implicated in EBV-mediated thyroid carcinogenesis, particularly in ATC. EBV has been closely associated with the loss of differentiation in thyroid tumors, promoting a shift toward more aggressive and treatment-resistant phenotypes. It is hypothesized that EBV infection may influence cellular differentiation states, thereby enhancing the malignant potential of tumor cells. This transition most commonly occurs at advanced disease stages or during tumor recurrence, when accumulated genetic alterations act synergistically with viral oncogenic effects. EBV infection accelerates this process through LMP1 and EBNA2 mediated EMT induction, tumor protein 53 (TP53) dysregulation, activation of NF-κB signaling, and remodeling of the immune microenvironment, thereby promoting undifferentiated and treatment-resistant phenotypes. Furthermore, EBV has been shown to affect the expression of tumor suppressor genes such as TP53 and neuroblastoma RAS viral oncogene homolog (NRAS), as well as genes associated with metastasis. These alterations contribute to malignant transformation and increased invasiveness. This effect is especially pronounced in undifferentiated thyroid cancer. LMP1 encoded by EBV plays a central role in this process by regulating the expression of metastasis-related proteins such as Nm23 and Twist, ultimately enhancing tumor cell motility and invasive behavior (92). In addition, EBV infection may promote angiogenesis, thereby supporting tumor growth and metastasis. Experimental evidence indicates that EBV can upregulate angiogenic factors, facilitating neovascularization within the tumor microenvironment. This pro-angiogenic effect further accelerates tumor progression and dissemination (93).

EBV infection induces a range of morphological and molecular alterations in thyroid tumor cells, with differential effects observed across various thyroid cancer cell lines. Experimental studies have shown that EBV triggers cytopathic effects such as localized monolayer degradation and cell fusion, particularly in the TPC-1 and 8505C cell lines. In contrast, no comparable cytological changes were detected in BCPAP cells. The replication kinetics of EBV also varied among cell lines. Both BCPAP and 8505C cells entered the viral “eclipse” phase at 24 hours post-infection. In contrast, TPC-1 cells exhibited low-efficiency viral production, with declining viral loads over time, suggesting a shift toward latent infection. At the molecular level, EBV infection significantly altered the expression of tumor suppressor genes such as TP53 and members of the Ras gene family. In BCPAP cells, both TP53 and NRAS expression were markedly upregulated. In contrast, while NRAS expression was also elevated in 8505C cells, TP53 expression was downregulated. These gene expression changes are closely associated with thyroid tumor progression, especially in highly aggressive subtypes such as ATC. These findings suggest that EBV may contribute to thyroid tumorigenesis by modulating key regulatory genes involved in tumor growth and malignancy (94). Immunomodulation represents another critical mechanism by which EBV influences thyroid carcinogenesis. The EBV-encoded ZEBRA protein plays a central role during the viral lytic cycle. ZEBRA can transcriptionally reprogram host immune regulatory genes, particularly those involved in T cell activation and cytokine signaling pathways. These immunomodulatory effects may impair local immune surveillance, promote immune tolerance, and create a tumor-permissive microenvironment that supports cancer cell survival and progression (95). EBV promotes thyroid cancer development and progression through multiple mechanisms, including immune evasion, chronic inflammation, tumor dedifferentiation, angiogenesis, and epithelial to mesenchymal transition. Its latent infection state, ability to modulate immune and molecular pathways, and impact on the tumor microenvironment offer promising avenues for future research in early diagnosis and therapeutic intervention. These findings also highlight potential molecular targets for the development of EBV-directed therapies in thyroid cancer.

#### 2.3.4 EBV in the transition from autoimmune thyroid disease to thyroid cancer

Persistent infection with EBV may promote malignant transformation of thyroid follicular cells by inducing chronic inflammatory responses. In patients with HT, sustained immune cell infiltration and continuous cytokine production create a pro-inflammatory and immunologically active microenvironment that favors EBV persistence and replication. This interaction between EBV and chronic immune stimulation may drive thyroid cell proliferation, genetic instability, and oncogenic transformation, potentially facilitating the transition from autoimmune thyroid disease (AITD) to thyroid cancer (90). One of the key mechanisms through which EBV contributes to this transition is molecular mimicry. The immune response elicited against viral antigens may cross-react with structurally similar thyroid self-antigens, resulting in immune-mediated tissue injury and sustained inflammation (51). During repeated cycles of inflammation and tissue repair, EBV can further activate oncogenic signaling pathways, reinforcing the progression from chronic autoimmunity to malignancy and positioning itself as a mechanistic bridge between inflammation and tumorigenesis. Supporting this, several studies have reported increased EBV positivity in PTC patients with underlying HT, lending credence to the role of EBV in inflammation-to-cancer transition (96). Mechanistically, EBV promotes the production of proinflammatory and pro-tumorigenic cytokines, such as Interleukin-6 (IL-6), tumor necrosis factor-alpha (TNF-α), and transforming growth factor beta (TGF-β), which in turn stimulate local cell proliferation, angiogenesis, and stromal remodeling. Concurrently, the virus enhances the infiltration of immunosuppressive cell populations, including regulatory T cells and M2-polarized macrophages, thereby establishing a tumor-permissive microenvironment that facilitates immune evasion (97, 98). Moreover, lytic EBV infection within tumor-associated macrophages has been shown to amplify inflammatory cascades and further reinforce immunosuppressive and pro-tumor mechanisms (99). In a study by Karaarslan et al., EBERs were detected via *in situ* hybridization in 58.06% of tumor cells, 53.2% of adjacent non-neoplastic follicular epithelial cells, and 16.1% of infiltrating lymphocytes in 81 PTC patients with coexisting HT. Notably, in patients with multifocal PTC, the EBV positivity rate in peritumoral lymphocytes was significantly higher (58.3%, p=0.034), suggesting a potential role for EBV in promoting multicentric tumorigenesis via immune microenvironment modulation within chronically inflamed thyroid tissue (100). Overall, the role of EBV in thyroid carcinogenesis extends beyond direct oncogenic activity and is intricately shaped by host immune status, human leukocyte antigen (HLA) background, chronic inflammatory burden, and environmental exposure. This process is multifactorial and dynamic, exhibiting strong population-specific patterns. These findings highlight the importance of continued investigation into the immunovirological interplay underlying thyroid cancer development in AITD patients.

### 2.4 EBV and other thyroid diseases

#### 2.4.1 EBV and subacute thyroiditis

Subacute thyroiditis (SAT) is an inflammatory thyroid disorder that typically presents with neck pain, transient thyrotoxicosis, and a self-limiting clinical course (101, 102). In recent years, viral infections have gained growing recognition as important triggers for SAT. Among the implicated pathogens are influenza virus, mumps virus, dengue virus, SARS-CoV-2, and EBV, all of which have been associated with SAT pathogenesis (103–105). Although EBV accounts for a relatively small proportion of SAT cases, several case reports support its potential pathogenic role. Volta et al. described a rare pediatric case of EBV-induced SAT, in which the patient developed markedly suppressed TSH, elevated FT3 and FT4, and increased inflammatory markers including C-reactive protein and erythrocyte sedimentation rate during an episode of infectious mononucleosis. Thyroid scintigraphy with 99mTc revealed markedly reduced uptake. Virological evidence included positive EBV DNA detection by PCR in both plasma and peripheral blood leukocytes, supporting a causal link between active EBV infection and SAT onset. The patient responded rapidly to a short course of glucocorticoids, and thyroid function normalized within a few months, indicating that EBV-related SAT is typically reversible and associated with a favorable prognosis (106). From a mechanistic perspective, EBV may contribute to SAT either by directly infecting thyroid tissue or by indirectly activating T cell mediated immune responses. These distinct processes can lead to follicular disruption and massive hormone release. As a consequence, transient thyrotoxicosis may occur. In addition, latent EBV antigens may interfere with apoptosis and antigen clearance, perpetuating local inflammation. Unlike Graves’ disease or Hashimoto’s thyroiditis, EBV-associated SAT is generally not accompanied by thyroid autoantibodies, suggesting that its pathogenesis is driven more by virus-induced immune activation than by classical autoimmunity (107). Clinically, EBV-related SAT may present with atypical or painless features, particularly in children or young adults, increasing the risk of misdiagnosis. In cases presenting with thyroid dysfunction alongside systemic features suggestive of viral infection, EBV testing should be considered as part of the diagnostic approach.

#### 2.4.2 EBV and thyroid adenoma

Thyroid adenoma is a monoclonal benign tumor originating from follicular epithelial cells. Although typically encapsulated and non-invasive, certain subtypes may exhibit premalignant potential (108, 109). In recent years, increasing attention has been directed toward the potential involvement of viral infections in the pathogenesis of thyroid adenomas. Among these, EBV has emerged as a particularly notable candidate. A study analyzing 133 thyroid adenoma specimens revealed an EBV DNA positivity rate of 37.6%, which is markedly higher than the baseline infection rate observed in the general population. This finding suggests that EBV may colonize or be transcriptionally active within adenomatous tissues (110). Mechanistically, although EBV primarily infects B lymphocytes, it can also infect epithelial cells under conditions of immunosuppression or local chronic inflammation. Latency-associated gene products such as EBNAs and members of the LMP family may promote clonal expansion by disrupting cell cycle regulation, enhancing cellular proliferation, and inhibiting apoptosis. Additionally, EBV has been shown to activate pro-inflammatory signaling pathways, including NF-κB, which may upregulate the expression of growth-promoting cytokines within the tumor microenvironment, further supporting adenoma development. The presence of an intact capsule in adenomas may limit immune cell infiltration and impair the clearance of infected cells, thereby facilitating viral persistence. Taken together, the presence of EBV in a subset of thyroid adenomas and its potential roles in promoting proliferation, inhibiting apoptosis, and mediating immune evasion suggest that EBV may act as a “facilitating factor” in benign thyroid tumorigenesis (111). These findings provide a rationale for further investigations into the virus’s contribution to adenoma molecular subtypes and the potential for EBV-targeted strategies in future personalized management approaches.

#### 2.4.3 EBV and thyroid Langerhans cell histiocytosis

Langerhans cell histiocytosis (LCH) is a rare clonal disorder originating from myeloid precursor cells (112, 113). It can involve multiple organ systems, including the bones, lungs, skin, and pituitary gland (114, 115). In rare cases, the thyroid gland may also be affected. A recent case study reported high EBV expression within a thyroid LCH lesion in a 29-year-old female. Both EBERs *in situ* hybridization and quantitative PCR revealed dense clusters of EBV-positive cells in the lesion, with significantly higher viral loads compared to adjacent normal thyroid tissue (p=0.022), suggesting a potential local pathogenic role for EBV in the formation of LCH lesions (116). Mechanistically, EBV may contribute to LCH pathogenesis by activating B cells, inducing Th1/Th17-mediated inflammatory responses, or establishing a locally immunosuppressive microenvironment that supports the proliferation and survival of aberrant Langerhans cells. Given the frequent presence of inflammatory cell infiltration in LCH lesions, EBV may act as a “second-hit” factor, exacerbating immune dysregulation and synergistically promoting lesion development. If future studies further substantiate the pathogenic involvement and molecular pathways of EBV in thyroid LCH, the virus may emerge as a potential therapeutic target. This could open new avenues for antiviral and immune-modulatory strategies in the management of EBV-associated LCH.

#### 2.4.4 EBV and Riedel’s thyroiditis

Riedel’s thyroiditis (RT) is a rare fibrosing inflammatory disorder characterized by extensive fibrosis of the thyroid gland and its surrounding tissues (117). Clinically, it presents with a painless thyroid mass, hoarseness, hypocalcemia, and, in severe cases, involvement of the recurrent laryngeal nerve. A case report described a 36-year-old female who initially presented with fever and neck discomfort. Early serological testing revealed EBV IgM positivity, indicating recent active EBV infection. The final diagnosis of RT was confirmed through surgical excision and histopathological evaluation (118). From a mechanistic perspective, EBV infection may act as a potential trigger for RT by activating chronic inflammatory pathways and promoting thyroid-specific autoimmunity, which in turn leads to progressive tissue injury and fibrotic remodeling. On one hand, EBV may stimulate the production of profibrotic mediators such as TGF-β and IL-6, which activate fibroblasts and enhance collagen deposition. On the other hand, recurrent episodes of virus-induced thyroiditis may establish chronic inflammatory foci, eventually culminating in fibrotic transformation. These processes may be particularly relevant in individuals with underlying genetic susceptibility to immune dysregulation. Although current treatment strategies for RT primarily rely on glucocorticoids and surgical intervention, the potential role of EBV in disease pathogenesis suggests that antiviral therapy or immune-targeted approaches may serve as adjunctive options in future clinical management (119).

### 2.5 Therapeutic potential of targeting EBV in thyroid diseases

Accumulating evidence indicates that EBV not only contributes to the onset of thyroid diseases but also plays a critical role in therapeutic responsiveness and recurrence risk. In Graves’ disease, preclinical studies have shown that EBV reactivation is linked to the generation of non-classical TRAb isotypes, which may compromise the long-term stability of therapy and increase relapse potential (120). Morgenthaler et al. further established EBV transformed B cell lines secreting both stimulatory and blocking TRAb isotypes, providing additional preclinical evidence that EBV actively drives pathogenic antibody diversity and interferes with treatment response (121). Experimental work has also demonstrated that EBV transformed B cells presenting thyroid peroxidase antigens can efficiently activate autoreactive T cell clones, offering a mechanistic basis for virus-driven immune amplification during therapy (122). In the clinical setting, case reports highlight that EBV associated hepatitis or systemic reactivation can precipitate autoimmune thyroiditis and aplastic anemia, with significant improvement following early immunosuppressive intervention (123). Collectively, these findings underscore the need to account for EBV related immune dysregulation when tailoring treatment strategies and evaluating prognosis.

In thyroid malignancies, EBV has emerged as both a potential oncogenic driver and a therapeutic modulator. Preclinical studies have documented upregulated expression of latent viral proteins such as LMP1 and EBNA2 in thyroid carcinomas, particularly in anaplastic thyroid carcinoma, where their expression correlates with epithelial–mesenchymal transition and increased invasiveness (124). Observational evidence from human tissue studies further indicates that elevated EBV DNA loads and EBER positivity in malignant thyroid tissues are associated with immune evasion and remodeling of the tumor microenvironment, potentially reducing responsiveness to radiotherapy and targeted therapies (82). Mechanistic experiments using thyroid cancer cell lines revealed that EBNA2 mediated activation of the NOTCH1 signaling pathway provides a direct link between viral latency and therapy resistance (125). Translational bioinformatics studies, including network pharmacology and molecular docking, have also begun to explore EBV targeted interventions; one such in silico analysis identified vitamin C as a repurposed agent capable of modulating EBV thyroid cancer co-expressed pathways through interactions with molecules such as LGALS3, MMP9, and CTSB (98).

Beyond malignancies, EBV has also been implicated in thyroid functional dysregulation during acute infection. Case-based observational evidence from Pariente et al. described a patient with severe hypothyroidism and paradoxical hormone profiles, suggesting that EBV may induce functionally active autoantibodies against thyroid hormones and thereby complicate biochemical monitoring and adjustments in replacement therapy (126). Taken together, these findings highlight EBV as an underappreciated determinant of therapeutic outcomes across thyroid disorders. By reshaping autoantibody repertoires, amplifying inflammatory cascades, and remodeling the tumor microenvironment, EBV may critically influence treatment efficacy. Nevertheless, the current literature is dominated by preclinical experiments, case reports, and small observational studies, while randomized controlled trials are entirely lacking. Future research targeting EBV through antivirals, immunomodulatory compounds or biomarker guided interventions will therefore be essential to provide higher level evidence for precision management of EBV associated thyroid diseases.

## 3 Discussion

EBV, a highly transmissible human herpesvirus with lifelong latent infection, has garnered increasing attention for its pathogenic roles in various autoimmune disorders and malignancies. This review outlines the potential mechanisms by which EBV contributes to the pathogenesis of diverse thyroid diseases, including GD, HT, thyroid cancer, and other rare thyroid conditions. By integrating serological, molecular, and histopathological evidence, we propose a sequential framework. It encompasses primary infection, viral latency, reactivation, chronic inflammation, immune dysregulation, and malignant transformation. For the first time, this longitudinal model maps a continuous pathogenic trajectory of EBV-driven thyroid disease development. Our findings suggest that EBV may function not only as a triggering factor but also as a pivotal pathological bridge and a promising therapeutic target within the thyroid disease spectrum.

In both GD and HT, EBV predominantly exists in a latent state. Numerous studies have identified EBV DNA or EBER-positive B lymphocytes in thyroid tissues and peripheral blood of affected individuals, while evidence of acute infection remains rare. This “quiescent yet reactivatable” infection pattern allows EBV to be reactivated under certain conditions, such as elevated estrogen levels, immune suppression, or concurrent infections. Upon reactivation, EBV enhances antigen presentation and promotes the expansion of autoreactive B cells (127, 128). In GD, EBV reactivation has been linked to abnormal production of TRAbs, driven in part by latent viral proteins like LMP1 and EBNA2, which promote B cell survival and immune activation via NF-κB signaling and CD40 mimicry (129–131). In contrast, in HT, EBV activates innate immunity through Toll-like receptor pathways, inducing Th1/Th17-driven inflammation, follicular damage, and autoantibody production. Importantly, the clinical outcomes of EBV infection appear to be shaped by host immune background, sex, infection timing, and the viral latency program (132, 133). For instance, in genetically susceptible female individuals, EBV reactivation more readily induces TRAb-IgM production, favoring a GD-like phenotype. Conversely, in hosts with a Th1-skewed immune response, the virus may drive chronic inflammation associated with HT. The pathogenic trajectory may also vary by latency type: EBER^+^/LMP1^-^ infections are more commonly associated with autoimmune phenotypes, while EBER^+^/LMP1^+^ infections are linked to tissue transformation and malignancy. EBV may also act as a molecular bridge between non-neoplastic thyroid autoimmunity and tumorigenesis. In patients with PTC arising in the context of HT, higher EBV positivity rates have been observed. Viral proteins such as LMP1 and ZEBRA activate metastasis-associated genes like Twist and MMP9 and upregulate anti-apoptotic proteins including Bcl-2 and survivin, promoting dedifferentiation and malignant transformation. Moreover, EBV reshapes the tumor microenvironment by enriching immunosuppressive populations, such as T regulatory cells and M2 macrophages. This weakens immune surveillance and facilitates cancer progression (134, 135). Therapeutically, the pathogenic involvement of EBV presents opportunities for targeted intervention. Disruption of EBV–B cell interactions or inhibition of the LMP1/NF-κB axis may help reduce virus-induced autoantibody production Preventive strategies targeting EBV reactivation, such as antiviral agents or immune-modulating compounds like vitamin C, may curb early disease progression. Recent studies also suggest that EBV-transformed B cells possess potent antigen-presenting capabilities, making their co-stimulatory signals and T cell activation pathways potential therapeutic targets. Furthermore, EBV-encoded markers may serve as promising biomarkers for disease subtyping and malignancy risk prediction, supporting precision diagnosis and personalized treatment strategies (92, 136).

While the immune evasive and oncogenic potential of EBV in thyroid diseases is well established, it is noteworthy that other oncogenic viruses such as human papillomavirus (HPV) and hepatitis B virus (HBV) also exhibit similar transitions between latent and lytic phases and possess immune remodeling capabilities (137). However, EBV is uniquely characterized by its ability to manipulate B cell biology and to persist within immunoprivileged environments like the thyroid, where it induces chronic antigenic stimulation (138). Compared to its role in nasopharyngeal carcinoma (NPC), EBV shares several common oncogenic mechanisms across both disease contexts, including LMP1 mediated NF-κB activation, antiapoptotic signaling, and evasion of immune surveillance (130, 139). Nevertheless, NPC typically arises from epithelial cells undergoing active lytic replication, whereas EBV in thyroid tissue is predominantly maintained in latency type I or II programs, suggesting a more subtle and immune mediated mode of action. This mechanistic divergence underscores the tissue specific behavior of EBV and highlights the possibility that its oncogenic role in the thyroid is more closely associated with chronic immune dysregulation rather than direct cellular transformation (140). Compared with previous studies, this work integrates the pathological connections between EBV and diverse thyroid diseases. It proposes a synthesized framework that organizes current evidence, highlights key mechanisms, and outlines directions for explicit, testable predictions. In addition, it offers detailed mechanistic insights into viral latency, B cell activation, immune regulatory networks, and remodeling of the inflammatory microenvironment. Despite the growing body of literature supporting an association between EBV and thyroid disorders, it is important to recognize that not all studies have reached concordant conclusions. Significant variability in EBV detection rates across geographic regions, methodologies, and patient populations raises concerns about the generalizability of current findings. Some case–control and serological studies have even failed to detect significant differences between patients and healthy controls. Moreover, most available studies are based on small cohorts, case reports, or cross-sectional designs, and the lack of standardized, sensitive detection techniques further introduces bias. These inconsistencies highlight the urgent need for large-scale, prospective, and multicenter studies to clarify the true nature and strength of the EBV–thyroid disease association. Future research should address these gaps by establishing large-scale, multicenter prospective cohorts to dynamically monitor the relationship between EBV infection and thyroid disease progression. Standardized and sensitive detection methods such as combining EBERs *in situ* hybridization, LMP1 immunohistochemistry, and qPCR viral load quantification are also needed to improve diagnostic reliability. In addition, therapeutic strategies targeting EBV latency, host receptors, or downstream pathways hold promise but require further validation. Integrative multi-omics approaches may help unravel the complex regulatory networks between EBV and the host, offering new insights into its pleiotropic effects. Overall, EBV may act as both a shared pathogenic trigger and a “molecular bridge” linking autoimmunity and tumorigenesis. Clarifying its roles in immune activation, tolerance breakdown, and tumor microenvironment remodeling will refine the virus–immune–thyroid axis and support the development of personalized antiviral interventions for earlier diagnosis, risk stratification, and precision management.
